# One arrow—two targets: Dual role of ETHYLENE INSENSITIVE2-like protein in osmostress and submergence tolerance

**DOI:** 10.1093/plphys/kiaf387

**Published:** 2025-09-01

**Authors:** Burcu Alptekin

**Affiliations:** Plant Physiology, American Society of Plant Biologists; Department of Bacteriology, University of Wisconsin, Madison, WI 53706, USA

Plants are highly responsive to the water in their surrounding environment. Different plant species have their sweet spot for the water they need, which is a construct of thousands of years of evolution, and positive and negative deviations from that sweet spot are undesirable for a plant. At first glance, dehydration and submergence in water are considered two contrasting water stresses; yet, they have overlapping consequences for plant function and survival (Chen et al. 2023). As an example, we can consider the two most common responses to these stresses. In response to dehydration, plants close their stomata to limit water loss, which in turn reduces the photosynthesis rate due to a reduction in CO_2_ intake. When plant roots are submerged in water, they cannot access enough oxygen, which triggers a transition from aerobic to anaerobic root metabolism. This shift in metabolism results in changes in plant metabolic activity and can even lead to root decay (Colmer et al. 2013). These two different responses to dehydration and submergence ultimately result in cellular damage, disruption of water and carbon fluxes, and, consequently, reduced growth or death of a plant. Therefore, it is expected that there will be some commonalities between dehydration and submergence stress responses at the molecular/cellular level. These common responses to both stresses include the formation of reactive oxygen species, the activation of an antioxidant defense system, and the initiation of molecular responses to different phytohormones (Renziehausen et al. 2024).

Recent work published in *Plant Physiology* shed light on the dual function of *ETHYLENE INSENSITIVE 2 (EIN2)*-*like* genes in the moss *Physcomitrium patens* in response to submergence and chemically induced osmostress with NaCl and mannitol (Karim et al. 2025). EIN2 is a central positive regulator in the plant ethylene signaling pathway (Alonso et al. 1999). Upon the perception of ethylene by histidine-kinase (HK) receptors localized in the endoplasmic reticulum (ER) membrane, EIN2 is cleaved and the EIN2 C-terminal end domain (CEND) localizes to the nucleus. Here, CEND stabilizes the ETHYLENE-INSENSITIVE 3 (EIN3) protein, leading to the activation of downstream transcription factors that promote ethylene responses (Wen et al. 2012). In *P. patens*, there are two EIN2-like genes, *PpEIN2A* and *PpEIN2B*, each encompassing a conserved N6-terminal transmembrane domain and a CEND region containing a putative nuclear localization signal (Karim et al. 2025). To functionally characterize the role of these genes in stress responses, the authors generated genome-edited indel mutants in *P. patens* for both *PpEIN2A* and *PpEIN2B*, as well as a double mutant of both, *ppein2ab*. Exposing the genome-edited lines of *P. patens* to ABA revealed that the double mutant line *ppein2ab* exhibits altered sensitivity to ABA. Wild-type *P. patens* showed inhibited growth and formed brood cells when exposed to ABA, a response typical of ABA sensitivity in mosses. However, *ppein2ab* lines were less sensitive to ABA and did not form brood cells. Further tests also showed that wild-type *P. patens* developed tolerance to osmostress after ABA or mild stress pretreatment, whereas this was not the case for *ppein2ab*.

Previous work from *P. patens* suggests an interesting connection between the regulation of submergence and osmostress tolerance in bryophytes through the activity of endoplasmic reticulum-localized ETHYLENE TRIPLE RESPONSE1-like histidine kinase (ETR-HK) and the B3-RAF kinase ARK (Toriyama et al. 2022). ETR-HK is upstream of B3-RAF kinase ARK and is required for its activation. Activated ARK further regulates the activity of SNF1-related protein kinase2 (SnRK2), which is an essential part of ABA signaling together with PYRABACTIN RESISTANCE1 (PYR)/PYR1-LIKE (PYL)/REGULATORY COMPONENTS OF ABA RECEPTORS (PYR/PYL/RCAR) and group A protein phosphatases 2C (PP2CAs) (Cutler et al. 2010). Toriyama and colleagues (2022) previously showed that disruption of either B3-RAF kinase ARK (*PpCTR1L*) or 4 ETR-HK genes results in the abolishment of ABA and osmostress-induced activation of SnRK2 in *P. patens*. In alignment with results from Toriyama et al. (2022), *ppein2ab* lines also showed reduced activity of SNF1-related protein kinase2 (SnRK2) upon ABA treatment, suggesting a connection between ethylene and ABA signaling pathways. Karim and colleagues (2025) also detected reduced expression of *LEA-like* genes, a common osmostress-responsive gene family, and reduced accumulation of LEA-like proteins in *ppein2ab* lines.

To further establish the connection between *PpEIN2* and ABA signaling, the authors examined ABA-induced gene expression with transient reporter assays using ABA-inducible promoters in wild-type *P. patens* and *ppein2ab* lines. Bombardment of protonema cells with GUS gene fused to the ABA-inducible Em promoter (proEm-GUS) and the luciferase (LUC) gene fused to the Ubiquitin promoter (proUbi-LUC) revealed that *ppein2ab* has a lower GUS:LUC ratio, suggesting a reduced ABA response compared to wild-type *P. patens*. Co-bombardment of *ppein2ab* protonema with PpEIN2A or PpEIN2B cDNA restored the ABA response, indicating that PpEIN2A or PpEIN2B are functionally redundant for the ABA response in *P. patens*. Authors also showed that the CEND domain of PpEIN2 is both necessary and sufficient for restoring the ABA response.

As another level of evidence for the connection between ethylene and ABA signaling pathways in *P. patens*, the authors investigated the possibility of phosphorylation of PpEIN2A by B3-RAF Kinase ARK (PpCTR1L) that was previously studied in a similar context by Toriyama et al. 2022. The authors used the *Escherichia coli*–expressed GST-fusion protein of the ARK/PpCTR1L kinase domain (GST-ARK-KD), which phosphorylates PpSnRK2B for in vitro kinase assays. Results from this assay showed that GST261 ARK-KD phosphorylates PpEIN2. These findings in ABA response and stress tolerance of *ppein2ab*, accompanied by results involving activity of SnRK2 and B3-RAF ARK kinase, suggest that *PpEIN2* is required for ABA-induced osmostress tolerance in *P. patens* (Karim et al. 2025).

Given the substantial evidence supporting the crucial role of ethylene in submergence, the authors also investigated PpEIN2 and PpEIN3, a gene located downstream of EIN2 in the canonical ethylene pathway, in the context of submergence stress. *ppein2ab* lines showed a defective response to submergence, similar to their response to osmostress. Wild-type *P. patens* exhibited branched protonemas in response to submergence stress (Yasumura et al. 2012), and this phenotype was absent in the *ppein2ab* lines. Authors quantified the expression of several submergence-regulated genes, such as *PpPIP2;2 and PpPIP2;3*. While the expression of *PpPIP2;2* increased and *PpPIP2;3* decreased in response to submergence in wild-type *P. patens*, the *ppein2ab* lines showed no change in the expression of these genes, supporting the phenotypic data and suggesting a malfunction in response to submergence. The results from *ppein3ab* mutants were different from those of *ppein2ab*. *ppein3ab* showed a defective response to submergence stress. In contrast, their osmostress and ABA treatment responses were similar to those of the wild type in terms of stress tolerance and growth inhibition. These results together indicate that PpEIN2 is not only essential for ABA-mediated osmostress responses but also may function as a key signaling component in ethylene-related submergence responses in *P. patens*. On the other hand, PpEIN3 is not involved in osmostress but is important for submergence response.

The *EIN2* gene is highly conserved across the plant kingdom, with homologs identified not only in angiosperms but also in more basal groups, such as the green alga *Chlamydomonas* (Qiao et al. 2009). In this work, Karim and colleagues demonstrated that *EIN2* orthologs may have a conserved role in osmostress responses across streptophytes, not just in *P. patens*. They tested the functions of *A. thaliana EIN2* (*AtEIN2*) and the EIN2 ortholog of *Chara braunii* (*CbEIN2*) in the ABA response to explore functional conservation of *EIN2* in streptophytes. Both *AtEIN2* and *CbEIN2* were able to restore ABA responses in *ppein2ab* lines, indicating evolutionary conservation of the protein itself. However, the ABA story of EIN2 in angiosperms is more complex. For instance, Arabidopsis *ein2* mutants exhibit either hypersensitivity or insensitivity to ABA, depending on the tissue type (Beaudoin et al. 2000). This difference might be due to more complex signaling networks that involve both ABA and ethylene responses in angiosperms, such as B3-Raf kinases. While *P. patens* has a single B3-Raf (ARK/PpCTR1L), *A. thaliana* has 6 (Katsuka et al. 2020). Some streptophyte algae lack EIN2 but retain other ethylene signaling components (ETR-HK, EIN3) (Ju et al. 2015), suggesting the EIN2-ABA connection evolved in specific lineages. Overall, the findings indicate that the ETR-HK–B3-Raf–EIN2 signaling module may have facilitated early plant adaptation to land by integrating responses to submergence and dehydration (Fig.). Further understanding this signaling module can help us find new ways to develop crop species that are flexible in their water response, adapting to both water scarcity and excess.

**Figure.**
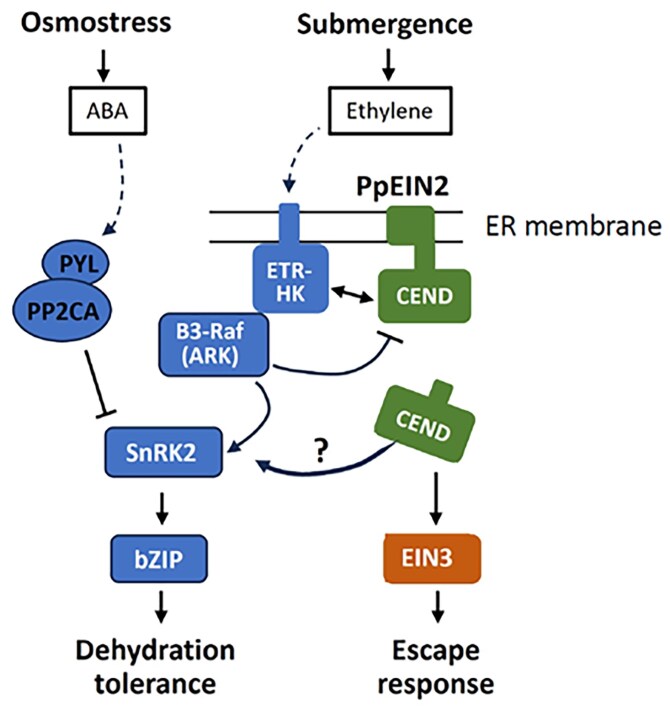
Proposed working model showing the roles of PpEIN2 in the regulation of osmostress and submergence responses in *P. patens*. While PpEIN2 mediates submergence signaling through the regulation of the EIN3 ortholog, it also affects the osmostress signaling pathway through the regulation of B3-Raf (ARK/PpCTR1L) and SnRK2.

## Data Availability

No new data were generated or analyzed in support of this research.
